# Biomechanical, biochemical, and morphological mechanisms of heat shock-mediated germination in *Carica papaya* seed

**DOI:** 10.1093/jxb/erw402

**Published:** 2016-11-02

**Authors:** Rachel E. Webster, Wanda M. Waterworth, Wolfgang Stuppy, Christopher E. West, Roland Ennos, Clifford M. Bray, Hugh W. Pritchard

**Affiliations:** ^1^Faculty of Life Sciences, University of Manchester, Oxford Road, Manchester M13 9PT, UK; ^2^Centre for Plant Sciences, University of Leeds, Woodhouse Lane, Leeds LS2 9JT, UK; ^3^Royal Botanic Gardens, Kew, Wellcome Trust Millennium Building, Wakehurst Place, Ardingly, West Sussex RH17 6TN, UK; ^4^School of Biological, Biomedical and Environmental Sciences, The University of Hull HU6 7RX

**Keywords:** *Carica papaya*, dormancy, germination, heat shock protein, seed coat, tropical crop.

## Abstract

*Carica papaya* (papaya) seed germinate readily fresh from the fruit, but desiccation induces a dormant state. Dormancy can be released by exposure of the hydrated seed to a pulse of elevated temperature, typical of that encountered in its tropical habitat. *Carica papaya* is one of only a few species known to germinate in response to heat shock (HS) and we know little of the mechanisms that control germination in tropical ecosystems. Here we investigate the mechanisms that mediate HS-induced stimulation of germination in pre-dried and re-imbibed papaya seed. Exogenous gibberellic acid (GA_3_ ≥250 µM) overcame the requirement for HS to initiate germination. However, HS did not sensitise seeds to GA_3_, indicative that it may act independently of GA biosynthesis. Seed coat removal also overcame desiccation-imposed dormancy, indicative that resistance to radicle emergence is coat-imposed. Morphological and biomechanical studies identified that neither desiccation nor HS alter the physical structure or the mechanical strength of the seed coat. However, cycloheximide prevented both seed coat weakening and germination, implicating a requirement for *de novo* protein synthesis in both processes. The germination antagonist abscisic acid prevented radicle emergence but had no effect on papaya seed coat weakening. Desiccation therefore appears to reduce embryo growth potential, which is reversed by HS, without physically altering the mechanical properties of the seed coat. The ability to germinate in response to a HS may confer a competitive advantage to *C. papaya*, an opportunistic pioneer species, through detection of canopy removal in tropical forests.

## Highlight

Dormant *Carica papaya* seeds germinate rapidly after a single heat shock, following seed coat weakening that requires *de novo* protein synthesis but is unaffected by the germination antagonist abscisic acid.

## Introduction

Dormancy is a block to germination, which persists in the imbibed state and represents an adaptive mechanism that enables a seed to delay germination until environmental conditions favourable for seedling establishment are encountered. Consequently, dormancy in different species can be broken by different environmental signals, commonly including light and temperature. The nature of the physiological and molecular mechanisms that underlie the switch from the dormant to the germinative state still remain to be fully delineated despite considerable progress made to date, mainly on model species (reviewed in Finch-Savage and Leubner-Meteger, 2006; Holdsworth *et al.*, 2008; Graeber *et al.*, 2012).

Dormancy is a property more often associated with temperate species, and the mechanisms that control germination in tropical ecosystems remain less well characterised. *Carica papaya* L. (papaya) is an opportunistic pioneer species native to tropical South America and is an important commercial crop of tropical regions worldwide. Seeds readily germinate when fresh from the fruit but germination is slow and erratic after desiccation (Wood *et al.*, 2000) or in the presence of the sarcotesta, which contains germination inhibitors (Lange, 1961; Chow and Lin, 1991).

Desiccation induces a state of conditional dormancy in *C. papaya*. However, high levels of synchronised germination of hydrated dormant seeds can be induced by brief ‘heat shock’ treatment, with an up-shift of 10 °C from an imbibition temperature of ∼25 °C proving most effective (Wood *et al.*, 2000). This upshift in temperature could be regarded not as heat shock *per se* but as exposure to higher temperatures naturally present in the climatic zone of *C. papaya*, representing a single alternating temperature pulse. Germination of ~70% of desiccated *C. papaya* seeds could be achieved by giving seeds a 2–6 h HS before returning them to ∼25 °C for relatively rapid and synchronous germination. In contrast, heat treatment had no effect on the dormancy behaviour of the desiccated *C. papaya* seed without prior imbibition, irrespective of variation in temperature and duration of treatment (Wood *et al.*, 2000). Thus, hydration is an absolute prerequisite as germination subsequently occurred only in seeds previously imbibed for 1–5 d prior to HS treatment.

Gibberellin has also been reported to significantly promote germination of papaya seeds. Exogenous application stimulates germination of both the fresh and dry seeds; and most studies have found increases in the total and rate of germination (Furutani and Nagao, 1987; Tseng, 1992; Andreoli and Khan, 1993; Paz and Vázquez-Yanes, 1998; Salomão and Mundim, 2000; Bhattacharya and Khuspe, 2001). Treatment with 200 ppm GA_3_ for 24 h raised the final percentage germination of fresh seeds from 10 different varieties of papaya but the scale of the increase remained variety-dependent (Bhattacharya and Khuspe, 2001). After 10 d drying at room temperature, a 24 h soak in 500 ppm GA_3_ was sufficient to stimulate 50% *C. papaya* seed germination, while 1000 ppm treatment yielded 90% germination (Tseng, 1992).

Very few species are known to germinate in response to HS as a germination trigger. A brief exposure to a higher temperature is also known to promote germination in seeds of *Mallotus japonicus* (Washitani and Takenaka, 1987), *Echinochloa crus-galli* (Taylorson and DiNola, 1989), and *Rumex obtusifolius* (Taylorson and Hendricks, 1972; van Assche and van Nerum, 1997). In these species there appears to be a physiological change in the seed induced by the temperature shift. For example, exposure of hydrated *E. crus-galli* seeds to 46 °C for over 24 h in the dark switched the dormancy of a proportion of the seeds from phytochrome-dependent to phytochrome-independent (Taylorson and Di Nola, 1989). Wood *et al.* (2000) suggested that as *C. papaya* seeds need to be hydrated for the temperature shift to be effective, then germination is also triggered by a physiological change, possibly involving protein synthesis and the activation of heat-sensitive signalling pathways. However, studies on other species conclude that HS proteins are unlikely to be involved (*R. obtusifolius*; van Assche and van Nerum, 1997) and that protein synthesis is not likely to be the key mechanism of sensing and responding to the heat treatment (*Cucumis sativa*; Amritphale *et al.*, 2000). Consequently, authors are using a variety of terms [e.g. ‘heat shock’, ‘temperature shift’, ‘brief high temperature exposure’ (Taylorson and Hendricks, 1972; Washitani and Takenaka, 1987; Taylorson and DiNola, 1989; van Assche and van Nerum, 1997; Wood *et al.*, 2000)] to potentially describe the same environmental trigger.

Other studies that have investigated the stimulatory effects of a brief temperature increase on germination have included the exposure of relatively dry seeds to fire. Usually the susceptible species possess hard protective outer tissues (seed coat or fruit wall) and the seeds are released from physical (water impermeability) or mechanical (restricted embryo) dormancy as heating ruptures these tissues (Vázquez-Yanes, 1974; Gashaw and Michelsen, 2002; Valbuena and Vera; 2002). In contrast, papaya seeds are readily water-permeable, even when dormant, and rehydration is essential to the stimulation of germination by HS treatment (Wood *et al.*, 2000). However, it is not known whether heat shock could act directly on the physical properties of the *C. papaya* seed coat to allow germination.

This study aimed to determine the mechanism by which heat shock mediates dormancy release in pre-dried and rehydrated papaya seeds. We show that physiological dormancy is coat-enhanced in nature, but that dormancy release is not accompanied by changes in the permeability, morphology, or mechanical properties of the seed coat. Stimulation of germination and seed-coat cracking by HS is shown to require protein synthesis but does not confer enhanced sensitivity to GA. These results point to critical roles for newly translated proteins in the control of papaya germination in response to rapid temperature fluctuations typical of unshaded gaps in the tropical forest canopy. This temperature-sensing mechanism thereby increases the potential for successful and rapid seedling establishment in this pioneer species, which is also an important commercial tropical crop.

## Materials and methods

Papaya (*Carica papaya* L.) fruit were kindly donated by the fruit importer Wealmoor Ltd (Jehta House, Springfield Road, Hayes, Middlesex, UB4 0JT, UK) and all fruit originated from the Caliman Agricola producer in Brazil. Seeds were removed from the fruit, the fleshy sarcotesta was removed, and then the seeds were dried in a climate-controlled room (~1 5°C and 15% RH), being placed in a monolayer on slatted trays. After 1 month, the seeds were transferred to open glass jars and stored under the same conditions. The batches of *C. papaya* seeds used in these experiments are described in Table 1. The moisture content of seeds and seed tissues was gravimetrically determined using oven-drying at 103 °C for 17 h (ISTA, 1999).

**Table 1.** Fruit and seed batch details. Means are shown with 95% confidence intervals. Moisture contents were determined gravimetrically from 10 replicates. All fruits were grown in the Sanctus Spiritus region of Brazil.

Characteristics	Batch 1	Batch 2
Variety	Golden Touch	Golden Touch
Number of fruits	106	96
Mean fruit mass (g)	313.9 ± 5.6	352.9 ± 4.1
Mean seed moisture content (%)		
Fresh	59.9 ± 0.8	59.1 ± 1.9
Washed	66.4 ± 5.7	68.4 ± 1.0
Dried	5.6 ± 0.4	5.1 ± 0.6

For the germination process, three stages were generally recorded: cracking of the seed coat (early germination), radicle emergence (completion of germination), and early seedling establishment based on adventitious root development and curvature (a ‘hook’) of the hypocotyl. Seeds at this early stage of seedling growth were judged to represent normal seedlings.

### Germination of de-coated, heat-shocked or gibberellin-treated seeds

Desiccated *C. papaya* seeds were imbibed for 5 d on 1% agar-water prior to removal of the seed coats under sterile conditions. Naked seeds were sown onto sterile 1% agar-water and HS treatments were given by moving seeds to a 35 °C incubator for 1, 2, or 4 h. Gibberellic acid (GA_3_) was provided to intact seeds at 100 μM and 250 µM in 1% agar-water, whilst the GA biosynthesis inhibitor tetcyclasis was provided at 1–100 µM in 0.1% acetone. Replicates of 3 × 25 seeds were sown in 9-cm diameter Petri dishes for all treatments. Imbibition and germination took place at 25 °C with an 8-h photoperiod (photosynthetically active radiation, PAR, 15–20 µmol m^–2^ s^–1^). Germination was recorded as radicle emergence over a 28-d experimental period. The mean time taken for seeds to germinate (in days) was calculated using the equation:

$$\text{Mean time to germinate }\left(\text{MTG}\right)\text{ =}\sum \left(N_{T}\times T\right)\text{/}\sum N$$

Where *N*_*T*_ = number of seeds germinated on day *T*, and Σ*N* = total number of seeds germinated during the experiment.

### *Treatment of* C. papaya *with germination inhibitors*

The potential germination inhibitors abscisic acid (ABA) and cycloheximide were applied to *C. papaya* seeds to test whether they could prevent the germination-stimulating effect of a HS. For the ABA treatment, desiccated seeds were imbibed on 1% agar-water containing 0, 10, 25, 50, or 100 µM ABA for 5 d and then received a 4-h HS at 35 °C. Seeds were transferred to fresh agar containing ABA after the HS. For the cycloheximide treatment, seeds were imbibed for 5 d on 1% agar-water before being transferred to filter paper soaked with 5 ml of water and 25 µg ml^–1^ or 50 µg ml^–1^ cycloheximide. After 2 h exposure to cycloheximide, the seeds were given a 4-h HS at 35 °C and then transferred to fresh cycloheximide-treated filter paper. Thereafter, the filter paper was moistened with 2 ml of solutions weekly. For the germination process, three stages were recorded as noted above.

### Cryostat sectioning and microscopy

Desiccated *C. papaya* seeds were imbibed for 5 d on 1% agar-water and then heat-shocked at 35 °C for 4 h. Imbibed (non-germinating) and cracked (germinating) seeds were selected to observe the changes occurring in the seed coat. Individual seeds were encased in Jung tissue freezing medium (Leica Instruments GmbH) and sectioned using a Leica CM3050 S cryostat microtome. Sections (20 µm thick) were adhered to glass slides prepared with Kaiser’s glycerol gelatine (Merck) and freeze-dried overnight at –20 °C. Slides were desiccated using a series of ethanol dilutions (50%, 70%, and 100%), covered with a drop of Histoclear (Sigma) to exclude air bubbles and mounted with Histomount (Sigma). Slides were studied on a Stemi SV11 (Zeiss) microscope and images were captured with a colour Axiocam (Carl Zeis Ltd.). Representative images were taken corresponding to transverse sections through the top (micropylar and embryo radicle end) and middle (embryo hypocotyl region) of the seed.

### *Biomechanics of the* C. papaya *seed coat*

Diametral compression tests were conducted on *C. papaya* seeds using a model 4301 universal testing machine (Instron). The Instron machine was adapted to perform a vice-like action with a hammer attachment. Seeds were placed such that the raphe ran parallel to the plates of the Instron attachments to ensure correct placement of the crack. The crosshead carried a 100 N load cell and the seed was compressed with a downward displacement of 5 mm min^–1^. The maximum load (N) borne by the seed coat at failure was recorded. Data from seeds found to be empty were excluded.

For analysis of seed-coat cracking on *C. papaya* germination, seeds were imbibed for 5 d before the seeds were manipulated by removing or cracking the seed coat. The seed coat was cracked by compressing the seed in the vice, placed with raphe running parallel to the jaws of the vice, until the seed coat ruptured. No adverse effects on endosperm or embryo integrity were observed.

### ^35^S labelling of *de novo* protein synthesis

Seeds were imbibed for 5 d at 25 °C on 1% agar-water before HS treatments. Seeds were de-coated (10 per sample) and incubated in 250 µl 50 mM sodium citrate/phosphate buffer (pH 6.8) with 150 µCi Trans^35^S-Label™ (ICN Biomedicals) and 0.1% Triton for 2 h at 25°C unless otherwise stated. Seeds were then rinsed with 2 l of water and protein samples were prepared by homogenisation in 250 µl chilled 50 mM HEPES buffer (0.6 mM DTT, 0.2 mM EDTA, and 0.03% Sigma plant protease inhibitor cocktail) and centrifuged for 5 min at 3500 *g* at 4 °C (Eppendorf 5417R centrifuge). Five protein samples were prepared with 2 h radiolabelling (1) pre-heat shock; (2) immediately after a 1-h HS; (3) during the 4-h HS (labelled at 35 °C during last 2 h of HS); (4) immediately after the 4-h HS (labelled after completion of the 4-h incubation at 35°C); and (5) 24 h after the completion of the HS.

Protein samples containing 100 000 cpm, as determined by scintillation counting, were separated by SDS-PAGE using pre-cast 4–12% bis-tris NuPAGE gradient gels (Invitrogen) (Laemmli, 1970). Proteins were blotted on PVDF using the XCell *Sure*Lock mini-cell system (Invitrogen). Dried membranes were exposed to X-OMAT film (Kodak) in an autoradiography cassette for 1–4 weeks and films were developed with X-OMAT developer and fixative according to the manufacturer’s instructions.

### Statistical analyses

Percentage germination data were normalised by arcsine square-root transformations before performing ANOVA using the statistical package SPSS.

## Results

### *GA_3_ and heat shock stimulate germination of the desiccated* C. papaya *seed*

**Fig. 1.**
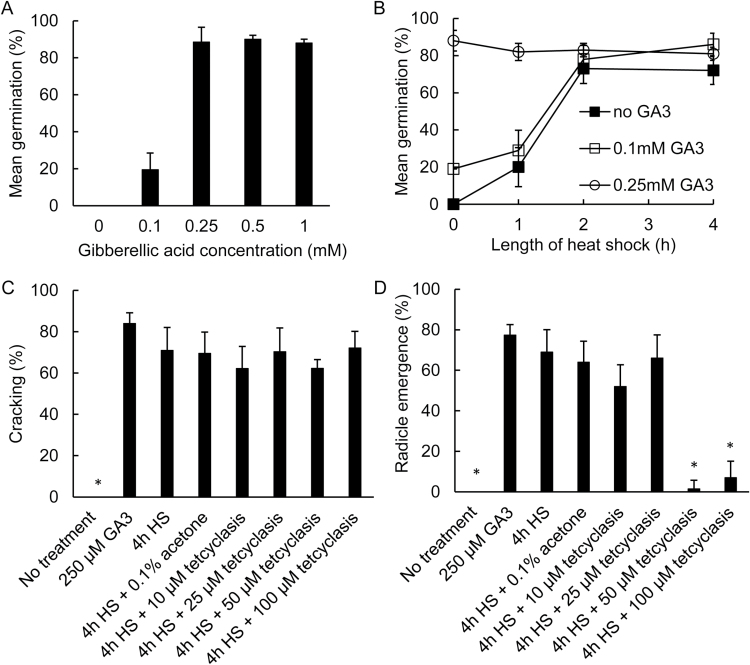
Effects of gibberellic acid and heat shock on germination of *C. papaya* seeds. (A) Effects of gibberellic acid on germination of *C. papaya* seeds. Seeds were sown directly onto agar containing up to 1 mM GA_3_ and imbibed at 25 °C. Error bars show SE, *n*=3. (B). Combined effects of gibberellic acid and heat shock on germination of *C. papaya* seeds. Seeds imbibed at 25 °C with 0.1 or 0.25 mM GA_3_ in 1% agar-water; controls are no GA_3_. Seeds were surface-disinfected after 5 d of imbibition at 25 °C and given a heat shock at 35 °C for 0, 1, 2, or 4 h before returning to 25 °C. Seeds were sown directly onto GA_3_ agar. Error bars show SE, *n*=3. (C, D) The effect of the the GA biosynthesis inhibitor tetcyclasis on heat-shock stimulated germination of *C. papaya* seeds. Mean percentage germination calculated at two stages: (C) initiation of germination (seed-coat cracking), and (D) radicle emergence (completion of germination). Standard 4-h heat shocks (HS) at 35°C were provided to seeds treated either with water, 0.1% acetone (v/v in water) or 0–100 µM tetcylcasis in 0.1% acetone. Error bars give 95% confidence inertvals. One-way ANOVA shows significant differences between treatments for both initiation (*F*_7,16_=48.66, *P*<0.0001) and completion (*F*_7,16_= 50.90, *P*<0.0001) of germination. (A–D) Final germination was scored at 28 d imbibition.

In initial studies we investigated the factors that stimulate germination in desiccated papaya seeds. Desiccation can induce conditional dormancy in *C. papaya* seeds and synchronised germination can be induced by HS treatment of the rehydrated seed (Wood *et al.*, 2000). In the current study we used batches of the papaya variety ‘Golden Touch’, which originated from Sanctus Spirtus province in Brazil (Table 1). Papaya seed germination occurs in three distinct phases: seed-coat cracking, radicle emergence, and seedling emergence. Surface-sterilisation of imbibed papaya seed had no effects on germination performance. Application of HS induced uniform germination in our experimental batches of desiccated *C. papaya* seed. Maximum germination was achieved when seeds imbibed for 5 d were treated with a 2-h or 4-h exposure to 35 °C before returning to 25 °C. Cracking of the seed coat (phase one), the first visible sign of germination, precedes radicle emergence through the endosperm (phase two), which occurs 1–2 d after seed-coat rupture for the majority of seeds. Within ~7 d of HS treatment adventitious root growth and hypocotyl curvature marks early seedling establishment (phase three). Pre-dried and rehydrated seeds did not germinate when imbibed on agar-water at 25 °C unless a HS treatment was provided (Fig. 1B).

Gibberellic acid is widely associated with stimulation of germination in a wide range of species. Accordingly, previous studies reported that *C. papaya* seed germination can be promoted by GA (Furutani and Nagao, 1987; Tseng, 1992; Andreoli and Khan, 1993; Paz and Vázquez-Yanes, 1998; Salomão and Mundim, 2000; Bhattacharya and Khuspe, 2001). Application of GA_3_ here also induced uniform germination in experimental batches of desiccated *C. papaya* seed, overcoming a requirement for HS (Fig. 1A).

The combined effects of GA_3_ and HS were evaluated next. Application of 0.1 mM GA_3_ stimulated about one-fifth of seeds to germinate, and a combination of 0.1 mM GA_3_ with HS of different lengths increased the total germination significantly by between 5 and 13% (Fig. 1B). However, in general, higher germination was attained by the single application of a longer HS of 2 h or greater (Fig. 1; two-way ANOVA: GA_3_ concentration *F*_1,16_=12.40, *P*<0.001; HS *F*_3,16_=59.44, *P*<0.001; interaction *F*_3,16_=2.16, *P*=NS). Approximately 80% of seeds germinated subsequent to treatment with 0.25 mM GA_3_ and at this higher concentration there was no improvement in total percentage germination when GA_3_ treatments were combined with a HS of any duration, thus indicating the total competence of the seed lot for germination. There was also no significant difference in total germination between non-heat-shocked seeds provided with 0.25 mM GA_3_ and water-imbibed seeds given a 4-h HS treatment (ANOVA: *F*_4,14_=0.91, *P*=NS). Imbibition of HS-treated seeds in the presence of the GA biosynthesis inhibitor tetcyclasis had little effect on seed-coat cracking, but exogenous application of 50–100 µm of tetcyclasis very effectively inhibited the completion of germination (Fig. 1C, D). HS therefore does not appear to sensitise seeds to GA_3_, and although GA biosynthesis is not evidently required for the stimulation of germination (seed-coat cracking) by heat shock, it is required for the completion of germination.

**Fig. 2.**
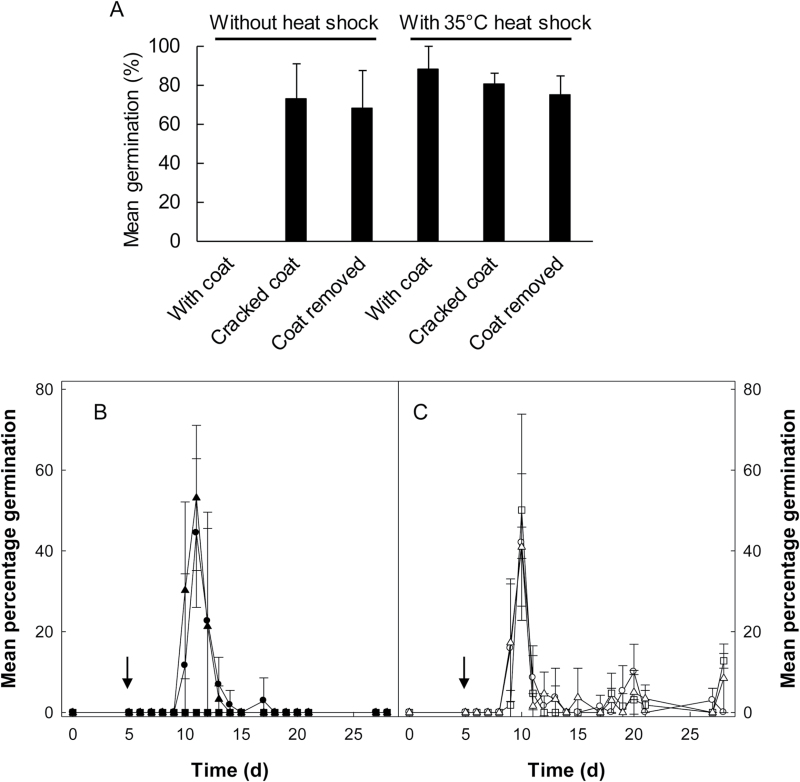
Effects of seed-coat removal on germination of *C. papaya* seed. (A) Germination of intact seeds, seeds with seed coats cracked using a vice (as described in the Methods), and de-coated seeds. Seeds were imbibed for 5 d at 25 °C and given a 0- or 4-h heat shock at 35 °C before returning to 25 °C. Data are shown with 95% confidence intervals. Final germination was scored at 28 d imbibition. (B, C). Rate of germination of naked *C. papaya* seeds. Intact seeds and de-coated seeds were surface-disinfected after 5 d of imbibition at 25 °C and given a 0-, 2-h or 4-h heat shock at 35 °C before returning to 25 °C. Mean germination is adjusted to account for damage caused during seed-coat removal. (B) Intact seeds (filled symbols) and (C) naked seeds (open symbols). Symbols denote heat shock treatments: (■ □), 0 h control; (● ○), 2 h; and (▲ △), 4 h. Data are shown with 95% confidence intervals. Arrows signify the timing of the heat shock application.

Dormancy can be associated with the embryo, endosperm, or the seed coat structures. Removal of the seed coat from *C. papaya* seeds (Fig. 2B, Table 2) or embryo excision (data not presented) also stimulated germination without HS treatment. Even cracking of the seed coat with a vice was sufficient to promote germination (Fig. 2A), indicating that dormancy in *C. papaya* is coat-enhanced in nature, and that release of dormancy by HS may act on the seed coat to release dormancy. The observation that seed-coat cracking can effectively promote germination suggests germination is not stimulated through counteracting germination inhibitors present in the seed coat. HS treatment to de-coated seeds did not improve the final percentage germination (ANOVA: *F*_2,6_=0.24, *P*=NS), with the peak of germination activity for both intact and de-coated seeds occurring soon after the HS/coat removal treatments performed on day 5 (Fig. 2C). This is suggestive that release from the seed coat can be substituted, but not improved, by HS treatment.

**Table 2.** Germination of seeds following seed-coat removal. Intact seeds and de-coated seeds were surface-disinfected after 5 d of imbibition and given a 0-, 2-, or 4-h heat shock. Data are shown with 95% confidence intervals.

Treatment	Heat shock length (h)	Mean germination (%)	Mean time to germinate (d)
With seed coat	0	0	–
	2	74 ± 2	11.5 ± 0.3
	4	87 ± 7	11.0 ± 0.3
Seed coat removed	0	68 ± 11	14.5 ± 1.6
	2	76 ± 4	12.4 ± 0.8
	4	75 ± 5	13.0 ± 3.3

### *Heat shock does not change the seed coat morphology of* C. papaya

The release of dormancy in *C. papaya* seeds by seed coat removal indicates that the seed coat could be the target site of action for the stimulation of germination by HS treatment and/or that the seeds undergo a physiological change in response to the temperature change. As *C. papaya* seeds require hydration for HS treatment to induce germination, this suggests that a physical change in the seed may also take place before germination occurs post-HS (Wood *et al.*, 2000). Initially, microscopy studies were used to identify if any visual changes in the physical structure of the seed coat were induced by HS treatment.

**Fig. 3.**
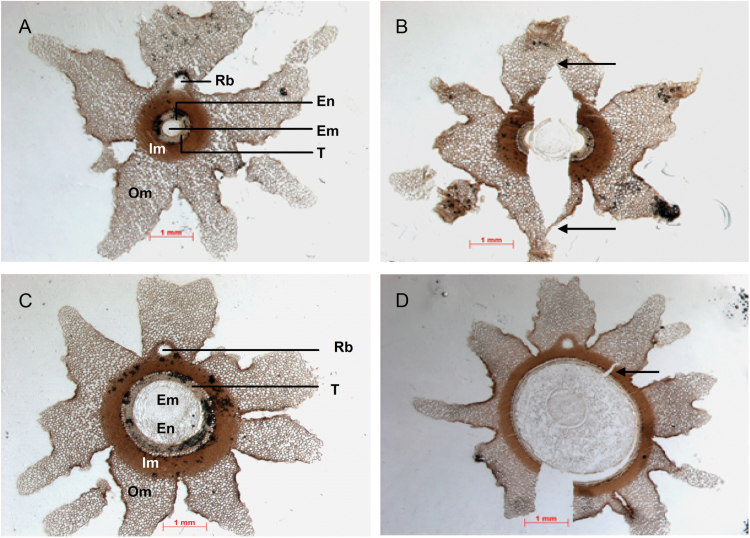
Effects of desiccation and heat-shock mediated germination on the morphology of the *C. papaya* seed coat. Intact seeds were surface-disinfected and imbibed for 5 d at 25 ° C before either no treatment or 4 h heat shock at 35°C and subsequent return to 25 °C. Transverse sections of dormant and germinating seeds. (A, C) Imbibed dormant seed (no heatshock); (B, D) germinating seed showing cracking of the seed coat (T: tegmen and endotesta) at 24 h post-heat shock; incomplete cracks are indicated by arrows. (A) and (B) are sectioned near the micropyle through the radicle; (C) and (D) are sectioned through the hypocotyl. Scale bars are 1 mm; 20-µm thick sections are magnified 20×. Em, embryo; En, endosperm; T, tegmen and endotesta; Im, inner mesotesta; Om, outer mesotesta; Rb, ovuolar vascular bundle contained within the raphe.

The *C. papaya* seed coat is a complex structure consisting of many layers derived from both the inner and outer integument (Corner, 1976). The pulpy sarcotesta forms the outermost layer of the seed coat and the mesotesta beneath is porous and spongy (Fig. 3A, C). The inner layers of the seed coat are harder and more brittle; the endotesta consists of a layer of crystal-containing cells and the tegmen is composed of a few layers of lignified cells (Corner, 1976). Transverse sections of a seed at the initial (cracking) stage of germination were analysed (Fig. 3B, D). The crack starts in the tegmen, near the endosperm, and runs into the spongy mesotesta. At the micropylar end of the seed (Fig. 3B) the two cracks have not completely broken the seed coat; they extend to the edge of the outer mesotesta. Further away from the micropyle (towards the centre of the seed, Fig. 3D), one crack has completely ruptured the coat while the other remains narrower and less extensive. Comparison of transverse sections from imbibed dormant and germinating seed (without HS and 24 h after HS treatment) do not reveal any visible differences in the structure of the layers of the seed coat. Analysis also identified little difference in the moisture content of the hydrated seed either before and after desiccation, or prior and subsequent to HS treatment, suggestive that neither desiccation nor HS act to alter the water permeability of the papaya seed coat.

### *Effects on heat shock on the mechanical strength of the* C. papaya *seed coat*

The effects of HS on the biomechanical strength of the *C. papaya* seed coat were next investigated. Studies of biomechanical changes in seed covering structures are often conducted by puncture tests, which are designed to replicate the rupture of these covering structures by the radicle. However, as cracking of the seed coat and endosperm puncture by the radicle during the germination of *C. papaya* are temporally distinct, these tests are inappropriate for studying seed-coat weakening. The first sign of germination of a *C. papaya* seed is the formation of a large crack running longitudinally along the seed and over the micropyle, which can be mimicked artificially by rupturing the seed coat with pressure from a vice. Consequently, the seed compression tests more often used to analyse the ability of a seed to withstand predation (Lucas *et al.*, 1991; Rodgerson, 1998) or gut-passage (Paulsen *et al.*, 2006) are appropriate for studying the strength of *C. papaya* seed coats during germination. During an ideal diametral compression test, the increasing load applied to the cylindrical test object creates internal tension stress until the strength of the object fails and a crack is induced along the diameter (Kamst *et al.*, 1999). In seeds, the internal tension created by external compression is considered to be the same as that created by hydraulic pressure from the seed contents (Paulsen *et al.*, 2006). Consequently, although the force applied by the external load (N) does not represent the force required by the embryo to escape, it has been used in ecological studies to calculate the work required to open the seed coat, simulating seed exit costs (Rodgerson, 1998; Paulsen *et al.*, 2006). Therefore maximum load (force applied at fracture) is highly correlated with work required to open the seed coat (Rodgerson, 1998).

**Fig. 4.**
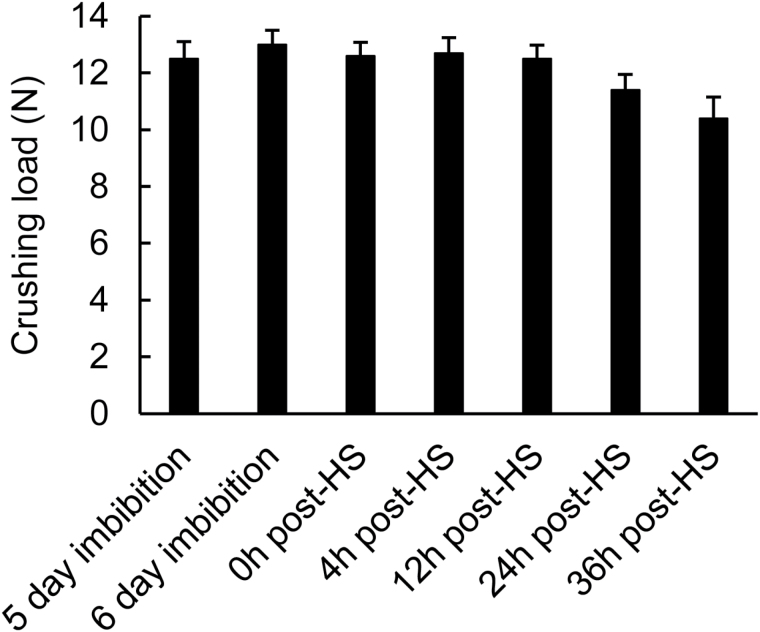
Biomechanical weakening of *C. papaya* seed coats prior to germination. Intact seeds were imbibed at 25 °C for 5 d, then a 4-h heat shock at 35 °C was applied, before returning the seeds to 25 °C. The 6-d imbibition treatment is equivalent to a non-heat-shock control. Seed-coat strength was measured by compression tests recording the maximum load carried before breakage. Error bars are SE; *n*=30. Seed-coat cracking associated with the progression of germination was observed at 48 h after heat shock.

In initial experiments to determine whether the seed coat altered following drying, seed-coat strength was compared between seeds freshly removed from the fruit (hydrated), desiccated seeds after 1 month dry storage, and seeds rehydrated for 1 d on 1% agar-water (Table 3; ANOVA, *F*_2,72_=190.82, *P*<0.001; 25 seeds crushed per sample). Seed coats were monitored using the maximum load (N) bearing capability of the tested material, enabling detection of the germination-induced changes and corresponding to a decrease in work required to escape the seed coat. Desiccated seeds could bear almost twice the load of hydrated seeds, but there was no statistical difference between fresh and 1-d imbibed seeds (Tukey pairwise comparisons, *P*>0.05), indicating that desiccation does not alter the biomechanical properties of the seed coat upon subsequent rehydration. The *C. papaya* seeds hydrated for 1 d could bear a load of approximately 13–14 N before the seed coats cracked. Changes in seed-coat strength during the time course of seed imbibition and HS treatment were then investigated. To test the effectiveness of the compression test at determining changes in seed-coat strength prior to germination, 30 seeds were crushed periodically up to 48 h after the HS. It took approximately 12–13 N to crack open the seed coat of papaya seed imbibed for 5 d (Fig. 4). The load required stayed the same up to 12 h after HS, but at 24 h and 36 h after HS the coats became significantly weaker (approaching 10 N required), as germination progressed to cracking of the seed coat. HS therefore does not appear to directly alter the mechanical properties of the seed coat either during or in the hours immediately subsequent to HS.

**Table 3.** A comparison of the seed coat strength of fresh, desiccated, and imbibed *C. papaya* seeds. Fresh seeds were removed from the fruit and cleaned, desiccated seeds were tested after 1 month of dry storage; imbibed seeds were placed on agar-water for 1 d at 25 °C. Seed strength was measured by compression tests recording the maximum load carried before breakage. The mean is shown with 95% confidence interval (*n*=25). * Means for desiccated seeds are significantly different to fresh or imbibed seed data (identified by Tukey’s pairwise comparison, *P*=0.05).

Seed state	Mean maximum load (N)
Fresh	14.2 ± 0.6
Desiccated	26.4 ± 1.5 *
Imbibed	13.4 ± 0.6

### Inhibition of germination by ABA

**Fig. 5.**
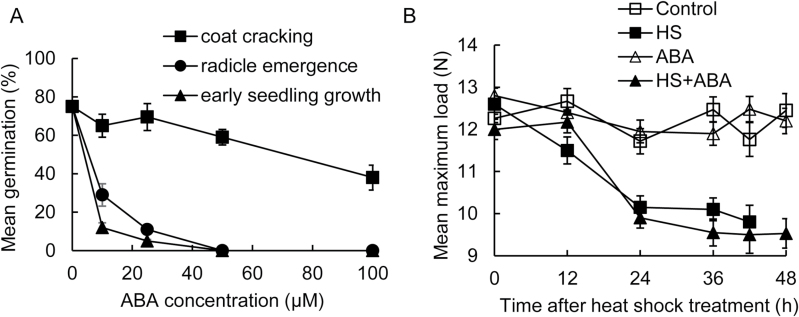
Effects of ABA on germination and mechanical strength of the seed coat in *C. papaya*. (A) Inhibition of *C. papaya* seed germination by ABA. Unless otherwise stated intact seeds were imbibed at 25 °C with up to 100 µM ABA for 5 d, then a 4-h heat shock at 35°C was applied, before returning the seeds to 25 °C. Seeds were imbibed continuously with ABA. Data show initiation of germination (coat cracking), completion of germination (radicle emergence), and early seedling growth. Error bars are SE; *n*=30. (B) Changes in strength of ABA-treated *C. papaya* seed coats prior to germination. Unless otherwise stated intact seeds were imbibed at 25 °C with up to 100 µM ABA for 5 d, then a 4-h heat shock at 35 °C treatment was applied, before returning the seeds to 25 °C. Seed-coat strength was measured by compression tests recording the maximum load carried before breakage. Key to symbols: (■ □), seeds imbibed on water agar; (▲△), seeds imbibed on 100 μM ABA agar; open symbols, no heat shock controls; filled symbols, seeds received 4-h heat shock at 35 °C. Error bars are SE; *n*=30. Seed-coat cracking was observed at 48 h after the heat shock in the water and positive heat-shock control; no germination was observed in other treatments.

As ABA is well established to play a role in the maintenance of seed dormancy and functions as a repressor of germination in many species, the interactions of ABA with *C. papaya* germination and HS treatment were investigated. *Carica papaya* seeds at different stages of germination responded differently to ABA (Fig. 5), possibly indicative that their sensitivity to ABA changes over the course of germination. Seed-coat cracking and radicle emergence displayed differential sensitivity to ABA. At the concentrations tested, exogenously applied ABA allowed over 40–70% of seeds to initiate germination as scored by visible cracking of the seed coat, but the majority arrested after the coat cracking stage (Fig. 5A). When supplied with ABA at higher concentrations of 50 and 100 µM, the radicle did not emerge from the endosperm and the seedling did not develop.

The effects of ABA on the mechanical strength of the seed coat after HS treatment were next investigated. Seeds were imbibed at 25°C on filter paper soaked with either water or 100 µM ABA for 5 d before receiving a 4-h HS at 35 °C. Seeds were then transferred to fresh water or ABA dishes after the HS treatment and returned to 25 °C. Whilst only 40% of seeds treated with 100 µM ABA and a 4-h HS displayed a visible seed-coat crack (Fig. 5A), biomechanical testing showed that these seeds weakened in a similar manner as the water controls (Fig. 5B). After a HS, seeds imbibed on both water-agar and 100 µM ABA show a significant ~2.5–3 N decrease in load-bearing capacity (two-way ANOVA: time after HS, *F*_4,300_=13.864, *P*<0.001; chemical, *F*_1,300_=14.192, *P*<0.001; interaction, *F*_4,300_=1.743, *P*=NS). At the later time points (36–48 h), seeds from the combined ABA and HS treatment were slightly weaker than those of the water control. Therefore, as ABA does not repress seed-coat cracking, but appears to repress radicle protrusion/embryo growth, we conclude that HS-mediated seed-coat weakening is independent of ABA. The action of ABA is not directly antagonistic to the mechanism of GA_3_ and HS action in release of papaya seeds from conditional dormancy.

### Inhibiting germination with cycloheximide

**Fig. 6.**
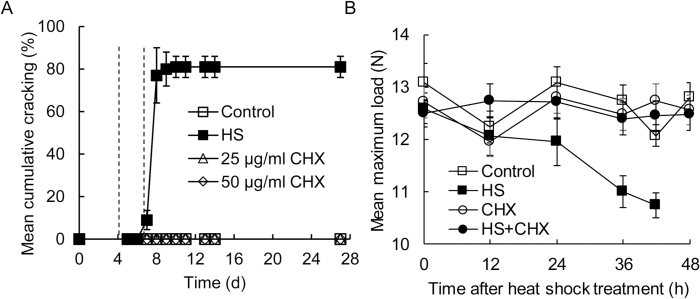
Inhibitory effects of the protein synthesis inhibitor cycloheximide on seed-coat cracking and biomechanical weakening of *C. papaya* seed. A. The inhibitory effect of cycloheximide application on *C. papaya* seed-coat cracking. Unless otherwise stated intact seeds were imbibed at 25 °C for 5 d, given a 4-h heat shock at 35 °C before returning to 25 °C. Symbols denote: (■), seeds imbibed on water that received a standard 4-h heat shock; (□), seeds imbibed on water with no heat shock treatment; (∆), seeds treated with 25 µg ml^–1^ cycloheximide (CHX) and given a standard heat shock; (◊), seeds treated with 50 µg ml^–1^ cycloheximide and given a standard heat shock. Treatments were applied at 5 d. Cycloheximide was provided 2 h prior to heat shock treatment to maximise uptake and throughout the remainder of the experimental period. Error bars give 95% confidence intervals; *n*=3. Biomechanical tests were conducted between days 5 and 7, as indicated by the vertical dashed lines. (B). The strength of the cycloheximide-treated *C. papaya* seed coat during germination. Key to symbols: (■ □), water controls; (● ○), seeds treated with 25 µg ml^–1^ cycloheximide; open symbols, no heat shock controls; filled symbols, seeds received 4-h HS at 35 °C. Treatments were given after 5 d of imbibition at 25 °C on water-agar. Cycloheximide was provided on filter paper 2 h before the standard heat shock treatment to ensure uptake into the seed and was also subsequently provided throughout the remainder of the experimental period. Seed-coat strength measured by compression tests recording the maximum load carried before breakage. Error bars give SE; *n*=30. Seed-coat cracking was observed at 48 h after the heat shock in the water and heat shock positive control; no germination was observed in other treatments.

As *de novo* protein synthesis is required for germination in a number of species, we next investigated the effects of a protein synthesis inhibitor on germination of desiccated *C. papaya* seeds. Application of cycloheximide prior to HS prevented germination of re-hydrated *C. papaya seeds.* Cycloheximide at concentrations of both 25 and 50 µg ml^–1^ prevented visible cracking of the papaya seed coat after HS treatment (Fig. 6A), implicating an absolute requirement for *de novo* protein synthesis in both seed-coat cracking and germination in *C. papaya*.

The dependence of seed-coat weakening on protein synthesis was next investigated through analysis of the effects of cycloheximide on the mechanical properties of the *C. papaya* seed coat (Fig. 6B). Following imbibition on 1% agar-water for 5 d at 25 °C, seeds were transferred to filter paper and imbibed in either water or 25 µg ml^–1^ cycloheximide for 2 h prior to a 4-h HS treatment at 35 °C. Seeds remained in contact with the cycloheximide during the course of the experiment. Non-heat-shocked control treatments were also supplied with water or cycloheximide throughout. Thirty seeds were used for each time point in the cycloheximide compression tests and seeds were crushed periodically up to 48 h after completion of the HS treatment. Seed cracking was observed in some treatments within the timescale of the experiments (48 h after HS), so time points that included visibly cracked seeds were excluded.

Fully hydrated seeds were treated with 25 µg ml^–1^ cycloheximide and subjected to HS treatments after 5 d imbibition before biomechanical tests were conducted over the following 48 h (Fig. 6B). All seeds imbibed for 5 d bore a maximum load of 12–13 N prior to coat rupture (Fig. 6B). Seeds imbibed in water as opposed to cycloheximide responded to HS treatments with significantly weakened seed coats prior to germination (ANOVA: *F*_4,150_=4.454, *P*<0.01). Seed coats were weaker at 36 and 48 h after the HS (one way Dunnet’s *t* at *P*=0.05 with the 0 h time point as the control group) and coats weakened by ~2 N by 48 h after the HS, while the strength of the non-HS control seeds remained relatively constant between 12 and 13 N. In contrast, when supplied with cycloheximide there was no seed-coat weakening irrespective of HS treatment (two-way ANOVA: time after HS, *F*_5,360_=2.443, *P*<0.05; chemical, *F*_1,360_=0.535, *P*=NS; interaction, *F*_5,360_=0.845, *P*=NS) (Fig. 6). Thus, inhibition of protein synthesis prevents seed-coat weakening and implicates HS in the induction of protein synthesis necessary for germination.

**Fig. 7.**
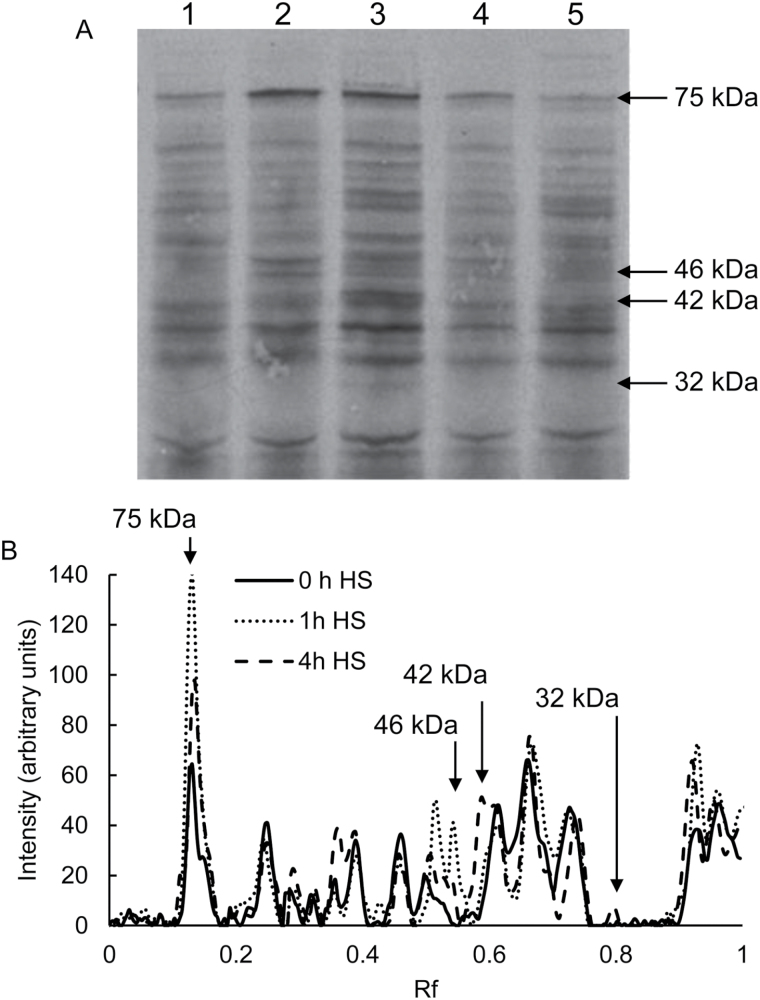
Rapid changes in *de novo* protein synthesis occur upon heat-shock treatment of *C. papaya* seed. (A) De-coated *C. papaya* seed were labelled with 150 µCi Trans^35^S-Label™ for 2 h (as described in the Methods) and extracted proteins were analysed by SDS-PAGE (4–12%) and autoradiography (100 000 cpm loaded per lane): Lane 1, protein extract from seeds imbibed for 5 d in water (no heat shock control); Lane 2, 1-h heat shock at 35 °C; Lane 3, during a 4-h heat shock at 35 °C seeds were incubated with label for the last 2 h of the heat shock; Lane 4, immediately after a 4-h heat shock at 35 °C seeds were incubated with label after completion of the heat shock; Lane 5, 24 h post-heat shock (which was the standard 4 h at 35 °C). Arrows indicate bands that alter over the time points. (B) Comparative profiles of Trans^35^S-Label™ incorporation into proteins separated by SDS-PAGE (4–12%) and analysed by autoradiography (100 000 cpm loaded per lane). 0h HS corresponds to Lane 1 in the gel presented in (A); 1 h HS corresponds to Lane 2; and 4 h HS corresponds to Lane 3. Arrows indicate bands that alter over the time points.

*In vivo* incorporation of ^35^S-methionine and ^35^S-cysteine was used to evaluate changes in protein synthesis in imbibed *C. papaya* seeds during seed treatments (Fig. 7). A number of changes in newly synthesised proteins were observable within 1–4 h from commencement of HS treatment (Fig. 7). The most prominent change in protein synthesis induced by HS treatment, as compared to the pre-HS control, was up-regulation of a 75-kDa protein (Fig. 7), which appeared most intense in seeds labelled 1 h after HS and seeds heat-shocked for 4 h (label provided within HS). A band of 46 kDa also appeared following 1 h of HS treatment, whilst subsequent to 4 h HS treatment bands of 76 kDa and 32 kDa appeared. A ~42 kDa band also displayed up-regulation after 1 h of HS, intensifying in the 4-h HS (label provided within HS) sample (Fig. 7).

## Discussion

Understanding the adaptive mechanisms that control timing of dormancy and germination is fundamental to crop propagation and the development of effective strategies to conserve biodiversity, but this remains less well characterised in tropical ecosystems. Here we characterise the mechanism by which brief exposure to a higher temperature, termed ‘heat shock,’ induces rapid germination in the tropical crop and opportunistic pioneer species *C. papaya*.

Desiccation induces a state of dormancy in *C. papaya* seeds, which otherwise germinate fresh from the fruit (Wood *et al.*, 2000). In agreement with a previous study, our data demonstrate that synchronised germination (≥70%) of desiccated papaya seeds can be induced either by ‘heat shock’ (4 h at 35 °C), or by exogenous application of GA_3_ (Wood *et al.*, 2000). The exposure of seeds to fire is often the focus of studies that investigate the stimulatory effects of a brief temperature increase on germination. Usually the susceptible species possess hard protective outer tissues (seed coat or fruit wall) and the seeds are released from physical (water impermeability) or mechanical (restricted embryo) dormancy as heating ruptures these tissues (Vázquez-Yanes, 1974; Gashaw and Michelsen, 2002; Valbuena and Vera; 2002). However, here we determine that although the *C. papaya* seed coat plays crucial roles in repression of germination, the morphological and biomechanical properties of the seed coat appear to remain unchanged by heat shock. *Carica papaya* seeds are readily water-permeable, even when dormant, and require full rehydration before the HS treatment is effective, indicative that a physiological change is necessary for this developmental switch from the dormant to the germinating state (Wood *et al.*, 2000). Furthermore, we find that heat shock-induced germination is absolutely dependent on *de novo* protein synthesis associated with the embryo and/or endosperm tissues.

Control of germination is based on a balance between the embryo growth potential and the restrictions of the surrounding tissues (Welbaum *et al.*, 1998), which in papaya includes the testa and the endosperm layer. Here, cracking or removal of the seed coat releases the block to germination, identifying that desiccation confers coat-enhanced dormancy on the seed. As removal of this mechanical barrier enables germination to proceed, this suggests that the embryos are restricted from growth by the seed coat. The reduced dormancy observed in a number of testa mutants identified the importance of the mechanical limitations imposed by the seed coat to Arabidopsis seed germination potential (Debeaujon and Kornneef, 2000). Although the load-bearing capacity of the seed coat increases upon desiccation, the mechanical properties of seeds when imbibed are similar to fresh seeds prior to desiccation, indicating that the coat is not mechanically changed by desiccation. The studies here suggest that HS does not act directly on the seed coat as no changes in seed coat morphology or mechanical strength are observed during, or in the first hours immediately subsequent to, HS treatment. If HS acts upon the seed coat to directly induce a mechanical change, seed-coat weakening should occur irrespective of seed capacity for germination. However, significant seed-coat weakening occurred in HS-treated *C. papaya* seeds only as they progressed towards germination. The protein synthesis inhibitor cycloheximide prevented both germination and seed-coat weakening, implicating an absolute requirement for *de novo* protein synthesis in both processes. *In vivo* ^35^S-methionine labelling studies demonstrated that changes in protein synthesis occur from 1 h after commencement of HS treatment. Our studies conclude that physiological changes must occur in the dormant *C. papaya* seed as it initiates germination.

The stimulation of *C. papaya* germination by seed-coat removal or cracking may be in large part attributable to the removal of the biomechanical barrier. Seeds are capable of overcoming this barrier when fresh from the fruit and germinate readily, but after drying this ability is lost and specific environmental conditions are required to enable escape from the seed coat. Desiccation appears to limit the growth potential of the embryo such that it cannot emerge after subsequent hydration and/or act to increase the resistance of the endosperm to rupture. The increase in embryo growth potential generated by the HS treatment is reliant upon *de novo* protein synthesis in the embryo and/or endosperm. As the germination crack is initiated in the inner layers of the seed coat, the observed weakening seems to result from the formation of small internal cracks reducing resistance to radicle protrusion. The dry seed coat tissues may not break as cleanly as hydrated tissues and so will require more force to tear apart. Cracks developing in wet shells of the African mongongo nut *Schinziophyton rautanenii* deviated around fibres whereas in dry shells the cracks tore across fibres, increasing apparent toughness (Williamson and Lucas, 1995).

Exogenous gibberellic acid can stimulate germination as effectively as heat shock treatment, but combinations of optimal GA_3_ concentrations and HS did not result in enhanced germination. Both treatments can effectively substitute for each other to trigger germination and seed-coat cracking was not inhibited by the GA biosynthesis inhibitor tetacyclasis, indicating that HS-mediated stimulation of germination is independent of GA biosynthesis. Our findings contrast with Arabidopsis (Nambara *et al.*, 1991), in which germination can be completely inhibited by GA biosynthesis inhibitors. Furthermore, studies with GA-deficient mutants indicated that GAs are necessary to overcome the restraint on germination imposed by the seed coat in Arabidopsis (Debeaujon *et al.*, 2000). The results of inhibitor studies must be interpreted with care as their effectiveness can be influenced by uptake efficiency and indirect effects arising from pathway disruption, such as the accumulation of intermediates (Redemacher, 2000). Gibberellin stimulation of *C. papaya* germination could substitute for light. However, the effectiveness of a HS treatment on *C. papaya* seed germination has been shown to be independent of the light conditions (Wood *et al.*, 2000), even though *C. papaya* seeds are capable of responding to light stimuli (Paz and Vazquez-Yanes, 1998). Wild papaya seed germination is inhibited by both darkness and an increase in far-red light, and although cultivation has reduced the sensitivity of commercial papaya seed to darkness, far-red light still inhibits germination (Paz and Vazquez-Yanes, 1998).

In species in which the covering endosperm structure obstructs radicle protrusion through the seed coat, such as papaya, early germination occurs in two distinct physical phases (Porceddu *et al.*, 2015). Initial tearing of the seed coat is followed by radicle protrusion through the endosperm. Our data points to differential regulation of seed-coat cracking and radicle emergence in *C. papaya*. ABA prevents radicle emergence but not seed-coat weakening, whilst studies with the GA biosynthesis inhibitor tetcyclasis suggest GA biosynthesis is required for radicle protrusion but not initiation of germination (i.e. seed-coat cracking). Thus application of exogenous ABA does not mimic the conditional dormancy state in *C. papaya*, as exogenously applied concentrations of 100 µM ABA permits seed-coat weakening and lower concentrations allow coat cracking. GA_3_ and ABA are well known for controlling embryo growth and endosperm weakening in several species, although species-specific differences are evident (Holdsworth *et al.*, 2008). ABA mediates at least partial inhibition of endosperm weakening in coffee (Amaral da Silva *et al.*, 2004), tomato (Toorop *et al.*, 2000), and Arabidopsis and *Lepidium* (Muller *et al.*, 2006), and also controls coffee embryo growth potential (Amaral da Silva *et al.*, 2004).

Cell wall modifying enzymes are important to germination by facilitating embryo cell expansion and endosperm weakening prior to rupture (Finch-Savage and Leubner-Metger, 2006). Studies have implicated endo-ß-1,3-glucanase, expansins, and endo-ß-mannose in endosperm weakening (Chen and Bradford, 2000; Muller *et al.*, 2006). Therefore, protein synthesis induced by HS in *C. papaya* may act to directly, or indirectly, trigger the synthesis of cell wall modification enzymes. Additionally, gibberellic acid is thought to stimulate germination either by inducing cell wall hydrolysis enzymes to weaken covering structures [e.g. *Lycopersicon esculentum* (Groot and Karssen, 1987); *Nicotiana tabacum* (Leubner-Metzger *et al.*, 1996)] or by increasing the embryo growth potential [e.g. lettuce (Takeba and Matsubara, 1979)].

Here, inhibition of protein synthesis prevents *C. papaya* seed germination at an early stage prior to the formation of visible cracks in the seed coat. Germination of other species including Arabidopsis is also established to be dependent on *de novo* protein synthesis (Rajjou *et al.*, 2004), although inhibition of transcription during imbibition of Arabidopsis seeds did not inhibit germination but did greatly influence the rate of germination (Rajjou *et al.*, 2004). Thus, successful germination of both *C. papaya* and Arabidopsis seeds is absolutely dependent upon *de novo* protein synthesis during imbibition and in Arabidopsis it appears that these proteins are synthesised from stored mRNA transcripts.

**Fig. 8.**
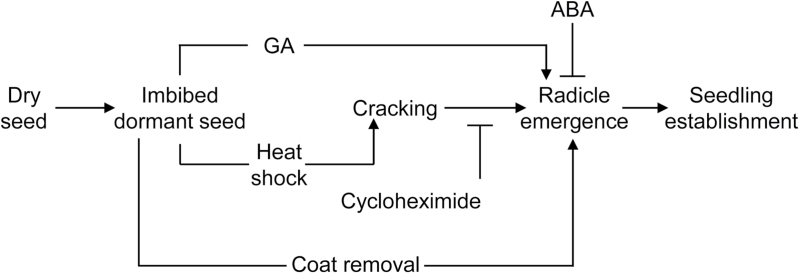
Model of germination stimulation and inhibition in imbibed *C. papaya* seeds subjected to heat shock and the biochemistry of the germination progress. Stimulatory treatments are indicated by →; inhibitory treatments are indicated by ┤.

In conclusion, the stimulation of germination in desiccated *C. papaya* seed germination by HS appears to result from physiological changes induced within the seed that are dependent on *de novo* protein synthesis. A schematic model of the factors controlling *C. papaya* seed germination is presented in Fig. 8. The embryo growth potential appears to be reduced by desiccation so that upon imbibition it has insufficient force to escape the mechanical restraint imposed by the surrounding structures. Germination of *C. papaya* seeds can occur if the seed coat is cracked or removed as the force imposed by the covering structures is reduced sufficiently so that it poses no significant barrier to the low growth potential of the embryo. Germination will also occur if there is a physiological change in the embryo or endosperm, which can be triggered by a HS or gibberellic acid, and which increases its growth potential sufficiently to overcome the physical barrier. The temperature shift required to ‘heat shock’ and induce germination in *C. papaya* equates to exposure to higher temperatures naturally present in the climatic zone where the species grows. In the natural habitat, gaps in the tree canopy can result in higher ground temperatures, stimulating germination. As *C. papaya* is an opportunistic pioneer species then the ability to germinate rapidly in response to a ‘heat shock’ may confer a competitive advantage in detecting canopy removal in tropical forests.
